# Developing a conceptual framework to facilitate inter-organizational partnership in disasters and public health emergencies

**DOI:** 10.1186/s12245-025-01027-7

**Published:** 2025-12-12

**Authors:** Jonas Zimmerman, Amir Khorram-Manesh, Eric Carlström, Yohan Robinson, Joakim Björås

**Affiliations:** 1 Institute for Clinical Sciences, Sahlgrenska Academy, Gothenburg University, Gothenburg, 405 30 Sweden; 2 Centre for Disaster Medicine, Gothenburg University, Gothenburg, 405 30 Sweden; 3 Gothenburg Emergency Medicine Research Group (GEMREG), Sahlgrenska Academy, Gothenburg University, Gothenburg, 413 45 Sweden

**Keywords:** Collaboration, Disaster, Emergency, Integration, Multiagency

## Abstract

**Introduction:**

Disasters and public health emergencies can overwhelm healthcare systems, requiring rapid increase in capacity, inter-agency collaboration, and the quick deployment of emergency medical teams. An effective response to these crises requires a structured approach that integrates multiple entities, including healthcare, military, and community organizations. Current frameworks often lack structured methods for optimizing collaboration, which can lead to redundancy and inefficiency. This study aims to address this gap by developing a conceptual framework combining collaboration and integration theories to improve inter-organizational partnerships in emergency management.

**Methods:**

To develop this framework, a comprehensive literature review was conducted identifying gaps in existing theoretical models of integration and collaboration. We examined integration theory at five distinct levels: structural, functional, normative, interpersonal, and process. Simultaneously, we explored collaboration theory, focusing on socio-cognitive, intersubjectivity, and distributed cognition frameworks. The study then synthesized these theories into a new, combined framework. This framework aligns integration components with key collaborative principles such as shared decision-making, effective communication, and adaptability. To make this framework a practical tool, we incorporated the CSCATTT model (Command and Control, Safety, Communication, Assessment, Triage, Treatment, Transport). This model typically used in collaborative exercises, serves as a practical guide for implementing and evaluating collaboration in real-world scenarios.

**Results:**

The proposed framework provides a structured approach for responding to disasters and public health emergencies by identifying key points where integration and collaboration can effectively be combined. It demonstrates that integration theory ensures system cohesion and operational efficiency, while collaboration theory fosters adaptability and stakeholder engagement. By aligning these two theories, the framework facilitates more effective multi-agency partnerships. The inclusion of the CSCATTT model enhances structured decision-making, enabling coordinated and efficient action during emergencies.

**Conclusion:**

By integrating collaboration and integration theories, this study offers a robust model for enhancing partnerships during disasters and public health emergencies. The proposed framework improves resource utilization, inter-agency coordination, cooperation, and overall effectiveness of collaborative responses, all while maintaining system flexibility. Future research should focus on validating this framework through practical applications, such as simulation exercises and real-world deployments.

## Introduction

The response to disasters and public health emergencies (DPHE) presents significant challenges to healthcare systems, often necessitating external support and deployment of emergency medical teams (EMTs). The core requirement is to guarantee victims a safe medical assessment at all levels of care. This calls for risk assessment, rapid decision-making, and a surge in capacity [1, 2]. Surge capacity refers to the ability of an emergency response system to rapidly expand its services and resources in response to a sudden increase in demand. It typically involves four key components, the so-called (4S): Staff, Stuff, Structure, and Systems [3]. Expanding surge capacity may include mobilizing additional personnel such as volunteers or retired professionals (Staff), securing stockpiles of essential resources and equipment (Stuff), repurposing facilities into treatment zones or establishing field hospitals (Structure), and ensuring efficient coordination through protocols and management systems (System) [3, 4].

Despite these measures, DPHEs are inherently unpredictable and dynamic, often stretching beyond the limits of conventional surge capacity. Responses are implicitly interdisciplinary, intradisciplinary, and multidisciplinary, involving coordination across agencies such as police, firefighters, healthcare, and, at later stages, actors such as nongovernmental organizations (NGOs) or the military [5]. When initial and secondary surge capacities prove insufficient, collaboration across multiple organizations becomes indispensable. Effective cooperation requires awareness of each entity’s limitations and strengths, beginning with coordination, followed by testing operational compatibility (cooperation), and ideally culminating in seamless collaboration [6, 7]. The aim is not organizational fusion but overlapping, prestige-free integration that combines adaptability with efficiency [5, 8]. Partnerships between healthcare, the military, and community actors have therefore been widely recommended [9, 10].

### The concept of flexible surge capacity

To address these evolving demands, the concept of Flexible Surge Capacity (FSC) has been introduced. FSC represents a novel, scalable, and adaptive approach to DPHE management, emphasizing the dynamic mobilization of healthcare and community resources beyond conventional planning frameworks [1]. It expands upon traditional surge capacity by integrating community-based, non-conventional, and rapidly deployable resources, thereby increasing resilience when standard healthcare systems are overwhelmed. Besides community integration and scalability of resources, its key features include proactive planning and multi-sector collaboration, particularly valuable in situations where conventional healthcare systems are insufficient [2, 11].

Rooted in complexity theory and collaborative models, FSC highlights the importance of resilience factors, adaptability, resource availability, and collaboration for preparedness and response [2]. Adaptability enables healthcare systems to adjust quickly to changing risks, resource availability ensures effective mobilization of supplies and personnel, and collaboration strengthens coordination among diverse stakeholders [12].

Although FSC has proven effective in enabling multi-agency responses, its core focus lies in collaboration. This highlights the need for a conceptualization advancement specifically the incorporation of integration theory into existing frameworks.

### Integration theory

Recent global and environmental changes, such as increased competition and technology-driven interconnectivity, have encouraged organizations to integrate. This renewed interest builds on decades of research, viewing integration along a continuum from coordination to cooperation, and ultimately collaboration [13]. According to Barki and Pinsonneault, integration is a process that creates a unified whole from distinct, interdependent components [14]. In healthcare, this ranges from ownership consolidation to improved care coordination. Singer et al. emphasize that integration is about creating connections across different parts of a system to facilitate interaction [15]. Healthcare’s complexity requires a precise conceptualization of integration’s components, which are commonly described as structural, functional, normative, interpersonal, and process integration.

Structural integration involves physical, operational, financial, or legal connections among teams and organizations within a health system. These connections can be horizontal (across similar organizations) or vertical (among different types of organizations and decision-making levels). Within organizations, structural integration may include creating clinical care teams or hiring staff such as care coordinators. However, having shared structures does not guarantee integrated activities, as some actions may occur spontaneously outside formal arrangements [15–17].

Functional integration refers to formal policies and protocols that coordinate activities and ensure accountability and decision-making among organizations and individuals. This includes mechanisms such as joint financial management, information management, strategic planning, and quality improvement. Unlike structural integration, which focuses on organizational arrangements, functional integration emphasizes standardized processes and is often under the direct control of leaders [17–21].

Normative integration emphasizes the sharing of a common culture that prioritizes coordinated patient care across units and organizations. It involves elements such as shared vision, mission, collective attitude, and leadership. This type of integration highlights the interaction between leadership and frontline norms and behaviors. While not directly controlled by leaders, their actions can strongly influence the degree of normative integration achieved ([22], Singer & Vogus, 2013).

Interpersonal integration concerns collaboration among healthcare professionals, non-professional caregivers, and patients. Unlike “professional integration,” which is limited to interactions among professionals, interpersonal integration includes patients and families, emphasizing their role in care. Effective interpersonal integration requires clear roles, positive teamwork attitudes, and strong relationships [21].

Process integration refers to coordinated actions that integrate patient care services across people, functions, and units over time. It includes activities such as shared care plans and communication of test results. While related to interpersonal integration, process integration focuses on translating collaboration into improved clinical pathways and outcomes. Leaders can facilitate this by providing supportive structures, functions, and communication tools [16, 18–20].

Mary Parker Follett earlier emphasized that integration is not only structural but also a social process that enables collaboration [23, 24]. Positive relationships and mutual respect are vital for consistent and coordinated work, and teamwork and collective norm development are essential for truly integrated care.

To comprehensively understand integration, it is important to distinguish between these different types. This also facilitates measurement. For example, structural and functional integration can be assessed through manager surveys, normative and interpersonal integration through staff surveys, process integration through administrative data, and contextual factors through market data [15]. Measurement tools should combine existing instruments with newly developed constructs, especially for interpersonal integration, which includes patients and caregivers. Using a mix of closed- and open-ended questions can provide a fuller assessment. Intermediate and ultimate outcomes may require multiple data sources, such as patient surveys for integrated care and typical indicators for health outcomes and costs. Integration theory provides the structural, functional, and social dimensions necessary to ensure system coherence however, these dimensions in themselves is not sufficient to effectively respond to the demands during DPHEs.

### Collaboration theory

Collaboration theory explores how individuals or groups work together to achieve common goals [25]. It encompasses various frameworks and models that explain the dynamics, benefits, and challenges of collaborative efforts. Collaboration is defined as a process involving two or more individuals working together to achieve a shared outcome. It can be synchronous (real-time interaction) or asynchronous (interaction over time). Collaborative learning strategies enhance problem-solving skills and social understanding [26]. Collaboration promotes a diversity of perspectives, which can lead to more innovative solutions. At the same time, effective collaboration requires clear communication and coordination, and mismatches in available resources and needs may constrain its success [25].

Several theories are particularly relevant for healthcare and teamwork. Socio-Cognitive Conflict Theory suggests that collaboration helps individuals resolve cognitive conflicts through interaction, resulting in improved understanding and problem-solving [27]. Intersubjectivity Theory emphasizes the shared understanding and mutual knowledge that develops during collaborative activities [28]. Distributed Cognition Theory highlights how cognitive processes are distributed across individuals, tools, and environments in collaborative settings [29].

Beyond theoretical models, Colbry et al. [25] developed a practical Collaborative Theory (CT) through the Farmer’s Exercise. Originally a physical strength exercise, it was used as a research scenario to observe how student groups communicated and worked together. Post-exercise interviews then provided qualitative insights into group dynamics. From this analysis, CT identified two broad categories of collaborative behavior. The first, “Individual First”, includes turn-taking, observing/doing, and status-seeking. The second, “Team First”, encompasses influencing others, organizing work, and building group cohesion. These categories illustrate that collaboration is not limited to leadership and followership but involves a dynamic interplay of individual and team behaviors that enhance performance.

In addition, Patel et al. [30] identified seven key factors influencing successful collaboration: (1) Context (task requirements, team type, support needed), (2) Support (adequate resources), (3) Tasks (clear definition of goals and roles), (4) Interaction process (communication, coordination, decision-making), (5) Teams (individuals with shared tasks and goals), (6) Individuals (whose performance is vital to success), and (7) Overarching factors such as trust, conflict, experience, incentives, constraints, and time. These factors highlight that collaboration is shaped simultaneously by individual, team, and systemic conditions.

### Collaborative factors and their role in facilitating integration and collaboration

In DPHE management, collaborative factors determine how effectively organizations can coordinate and respond. These factors include the context of the incident, the availability of support and resources, clearly defined tasks, team structures, individual contributions, and overarching conditions such as trust, conflict, goals, incentives, and time [30]. Effective collaboration also requires learning, communication, and mutual understanding before decision-making. When these factors are considered alongside different types of integration, it becomes clear that DPHEs typically involve all integration types interchangeably, with several items recurring during the process [2, 11, 12].

Table 1 illustrates how the five integration types, structural, functional, normative, interpersonal, and process can be aligned with collaborative elements, and how this alignment can be operationalized using the CSCATTT tool. The acronym CSCATTT stands for Command and Control, Safety, Communication, Assessment, Triage, Treatment, and Transport, and it represents the necessary steps in DPHE management.

**Table 1:** Mapping Integration Types to Collaboration Elements and the CSCATTT Framework

Type of integration	Integration theory	Collaborative theory	Collaborative tool	CSCATTT
Structural Integration (Horizontal and vertical organizations)	Physical, operational, financial, or legal connections: leadership, team composition, interoperable information systems, and governance mechanisms	Context Support Teams	Command & control Communication Assessment	C C A
Functional Integration	Formal policies and protocols that coordinate activities and support accountability and decision-making among organizations and individuals	Tasks Interaction process Overarching factors	Command & control Safety Communication Assessment Triage Treatment Transport	C S T T T
Normative Integration	Sharing a common culture that prioritizes integrating patient care across units and organizations. It involves shared vision, mission, collective attitude, and leadership	Overarching factors Teams Individuals	Assessment (situational awareness & Cultural considerations) Command & control	A C
Interpersonal Integration	Collaboration among healthcare professionals, non-professional caregivers, and patients. Clear roles, positive attitudes, strong relationship	Teams Individuals Tasks Context	Command & control Communication	C C S
Process integration	Coordinated actions that integrate patient care services across people, functions, and units over time. Focuses on translating collaboration into improved clinical processes	Teams Individuals Overarching factors Context Support	Command & control	C C A T T T

The CSCATTT framework is closely linked to the Major Incident Medical Management and Support (MIMMS) course, established in the UK in 1994. Its principles provide a structured, systematic approach to managing major incidents, ensuring complex tasks are broken down into manageable components. Over the past three decades, CSCATTT has been widely adopted in both civilian and military healthcare, and its principles have been incorporated into NATO medical training directives [31]. By structuring command, safety, communication, and clinical processes, CSCATTT enables agencies to plan and evaluate interagency collaboration more effectively, reducing redundancy and enhancing coordination [32–34].

Altogether, linking collaborative factors to integration types, and operationalizing them through CSCATTT, provides practical “touch points” for agencies to work together during emergencies.

The following section of the article aims to:

- Review the current state of collaboration and integration in emergency response.
- Propose a refined concept through a clearly defined methodology.
- Outline the implications of this novel approach for future emergency preparedness and response strategies.

## Method

### Design

This study employed a critical integrative review methodology to explore and construct a conceptual framework for collaboration and integration within emergency response systems. The integrative review approach was chosen for its ability to synthesize diverse forms of evidence, including empirical studies, theoretical literature, and policy documents across disciplines and methodologies. This flexibility is essential for identifying gaps in current systems and for addressing the complexity and evolving nature of multiagency coordination in emergency contexts [35]. The review process was conducted iteratively: first to identify existing theories and gaps, and subsequently to refine the proposed conceptual framework after its initial formulation. Integrative reviews of this type support the inclusion of both quantitative and qualitative studies, as well as conceptual and theoretical contributions, enabling the development of new insights and frameworks [36].

### Search strategy

A structured literature search was performed across three major databases: PubMed, Scopus, and Web of Science. These databases were selected for their complementary strengths in indexing biomedical, multidisciplinary, and high-impact literature. The search focused on publications from 2019 to 2025, a period marked by the COVID-19 pandemic, which not only exposed vulnerabilities in existing emergency response systems but also catalyzed significant advancements in collaboration, integration, and system design. Keywords included “collaboration,” “integration,” “emergency response,” “disaster management,” and “multiagency coordination.” Boolean operators and filters were applied to refine the results and ensure relevance to the study objectives. Two reviewers independently screened titles/abstracts and full texts against predefined criteria. Records were de-duplicated prior to screening. Disagreements were resolved by discussion; a third reviewer arbitrated when needed.

### Inclusion and exclusion criteria

Studies were included if they addressed collaboration or integration in emergency or disaster response contexts, were peer-reviewed, published in English between 2019 and 2025, and presented empirical findings, theoretical models, or conceptual discussions. Studies were excluded if they focused on unrelated fields, lacked relevance to emergency response, or were non-scholarly or lacked methodological transparency.

### Data extraction and analysis

A content analysis was performed using NVivo software, commonly used for qualitative data analysis (QDA) that helps to organize, analyze, and find insights in unstructured data like interviews, surveys, social media, and more [37]. Coding combined deductive and inductive approaches: directed coding was guided by a priori constructs from integration and collaboration theories, while inductive expansion was applied when new categories emerged. The process incorporated critical reflection to evaluate existing models and to identify opportunities for conceptual advancement.

### Concept development

Based on the synthesis of the included studies, a conceptual framework was constructed (described in detail in results section) to illustrate the key dimensions and interrelationships of collaboration and integration in emergency response. The framework was designed to combine theoretical insights from integration theory and collaboration theory with practical tools such as CSCATTT, thereby providing both a conceptual and operational foundation for multiagency coordination in DPHE management.

## Results

### Study selection

In total 105 papers were reviewed for eligibility (PubMed = 20; Scopus = 44; WoS = 41). The final number of papers included in the review was 21.

### Synthesis of the included studies

The studies collectively emphasize that effective disaster preparedness and response are not isolated activities but depend on an integrated, multi-layered, and collaborative ecosystem. The key to resilience lies in moving beyond traditional, top-down structures to embrace networked approaches that involve all levels of society, supported by robust data systems and a focus on equity. Using NVivo software, four overarching themes were identified.

#### Theme 1: Collaboration is the cornerstone of effective response

Collaboration and coordination are foundational requirements for managing complex crises [38, 39]. Internationally, this is seen in cross-regional efforts for pandemic preparedness, such as the ECLIPSE consortium’s work on surveillance and data sharing [40]. Domestically, events like terror attacks demonstrate the need for seamless cooperation between agencies such as police services, prehospital agencies and hospitals. Vertical, siloed structures often impede coordination, while horizontal arrangements facilitate information sharing [41] and the creation of joint command centers and exercises [42]. Barriers such as differing motivations and leadership styles may hinder cross-sector collaboration [43]. To address these challenges, conceptual frameworks such as the 3 C Model (Communication, Coordination, Cooperation) have been developed to analyze how technology can support collaboration [44].

#### Theme 2: Shifting from top-down management to community engagement and equity

A second theme highlights the shift from centralized, agency-led responses toward approaches that actively integrate and empower communities. Effective planning requires engagement with racially and ethnically diverse communities, who often face disproportionate burdens, by addressing barriers such as language, trust, and socioeconomic status [45, 46]. This involves moving toward community-led approaches that value local knowledge and non-official groups, recognizing community members as active participants (“steerers and rowers”) rather than aid recipients [47, 48, 49]. This sentiment is echoed in the call to balance external Emergency Medical Teams (EMTs) with the empowerment of local communities [50]. A practical method for this is disaster citizen science, where public participation in scientific activities can build capabilities in surveillance and volunteer management [51].

#### Theme 3: The critical role of data, surveillance, and shared understanding

A third theme emphasizes that effective decision-making in crises depends on strong information systems. Infectious disease threats highlight the need for integrated surveillance platforms and enhanced data sharing, which are also essential in humanitarian crises [40, 52]. To ensure all responders have a shared understanding, various tools and frameworks can be utilized to create a “system-level common operating picture” for real-time, multi-agency decision-making [53]. Studies also point to the importance of robust IT systems to manage evolving collaboration networks and resource flows during disasters [54].

#### Theme 4: Building system resilience and flexible capacity

Finally, hospitals and health systems must develop resilience and adaptability to withstand the stress of major crises. Simulation-based clinical systems testing (SbCST) has been used to identify latent safety threats before emergencies [55]. The concept of FSC provides a scalable way to mobilize community resources when conventional healthcare is insufficient [2]. Resilience is further supported through measures as WASH (Water, Sanitation, and Hygiene) services, vaccination campaigns, and integrating immunization into health services during humanitarian crises in low- and middle-income countries [52]. Another example is incorporating post-exposure prophylaxis (PEP) into mass casualty protocols [56].

### What is Missing: The gaps and issues

Despite established research and frameworks, the complexity of DPHE management continues to pose challenges [57, 58]. A recurring issue is the insufficiency of surge capacity, requiring expansion with community resources, paramedical specialties or military support [11]. While FSC aims to address this gap, it may also add to system complexity, underscoring the need for new approaches in education, training, and partnership [2, 11, 59].

A further gap remains in the practical application [60]: although partnerships between diverse organizations are frequently mentioned, there are few, if any, frameworks detailing how these entities should work together to maximize impact without duplicating efforts and wasting precious resources. This is particularly evident in the context of civil-military interactions. A recent study identified key facilitators highlighting shared experience and resources but also pointing out significant constraints. Mismatch between the needs in a crisis and available assets could severely impact the effectiveness of these collaborations. This underscores the importance of careful analysis, understanding the consequences, potentials, and limitations of partnerships, especially given the rise in global conflicts and constrained healthcare resources [12]. Similarly, limitations in community involvement for disaster risk reduction are noted, linked to relational and structural barriers [61].

### Enhancing integration and collaboration

By training and involving multiple agencies in DPHE preparedness, swift action can be taken to better manage immediate needs. Trust between responders and affected populations enhances cooperation and acceptance of required interventions [62, 63]. Strong leadership, decentralized decision-making, and continuous training ensure responders' readiness to act when needed [64–67]. Infrastructure and technology are foundational to effective response and addressing mental health needs increases coping with the stress of disasters [68, 69]. Finally, strengthening local health systems and focusing on long-term recovery ensure communities sustain their health services and rebuild effectively. By integrating these resilience factors into DPHE planning and response, systems become more adaptable, efficient, and capable of handling immediate and long-term challenges [70].

Generally, and specifically in some countries, the partnership between healthcare, the military, and the community is an essential first step to increase resilience and response skills. The World Health Organization (WHO) advocates strengthening of health systems through community empowerment and preparedness training [71]. Building local capacity, fostering cross-sector collaboration and improving health data systems are key strategies for enhancing resilience. Education, active participation, and equitable resource accessibility foster trust and partnership. Assessing potential threats, and mitigating their impacts are foundational steps toward achieving community resilience [72]. WHO recommendations emphasize the importance of flexibility and community involvement in DPHE response and collaborative partnerships with entities such as EMTs [73]. Given the evolving nature of emergencies, a more adaptive and integrated approach is needed to leverage synergies to enhance response effectiveness and promote long-term resilience [11, 74]. Simulation exercises are also recommended to increase inter-, intra-, and multidisciplinary collaboration between organizations and enhance DPHE response effectiveness [34].

### Developing an integrative-collaborative conceptual framework for DPHE management

#### Core partnership and organizational ecosystem

**Fig. 1:**
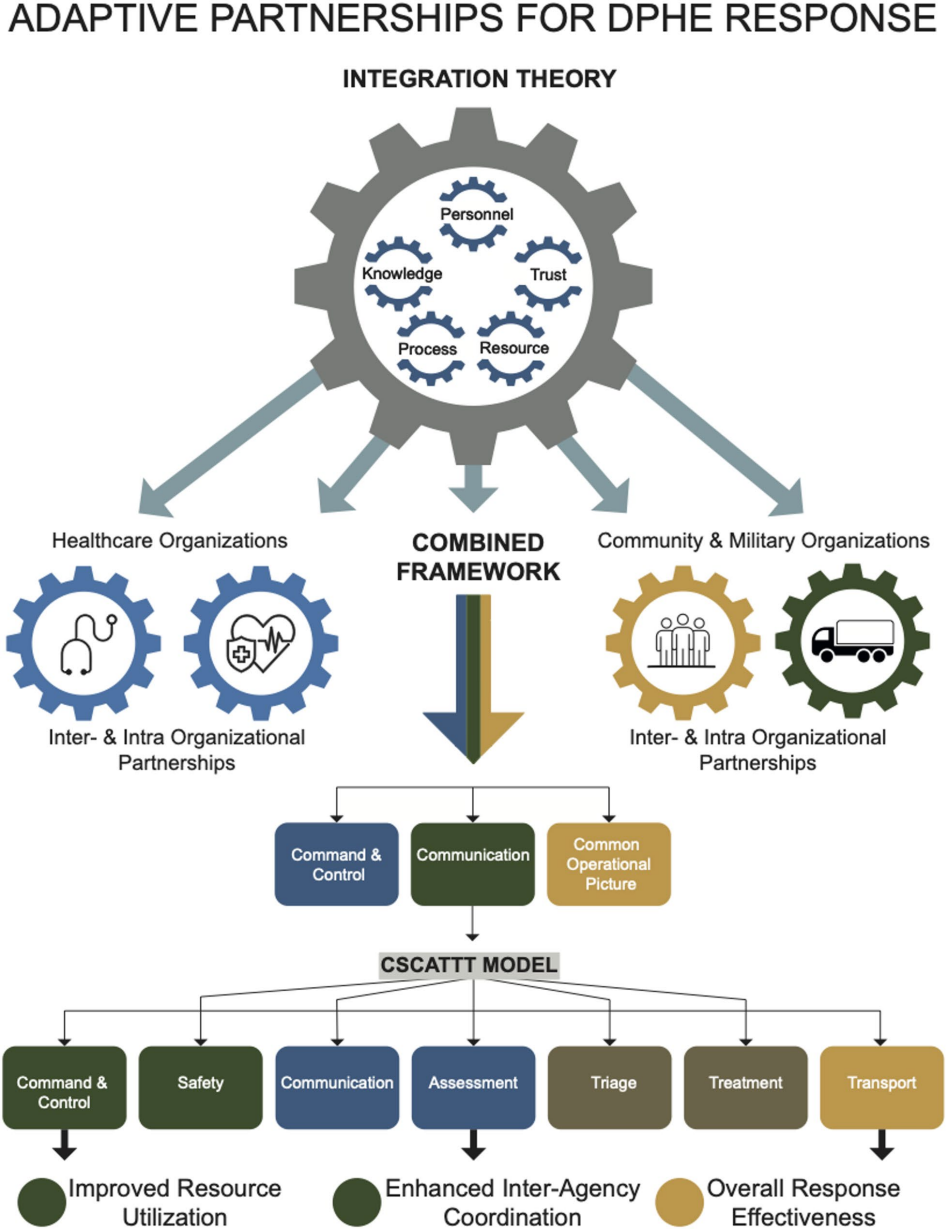
The Core Partnership At the top, the integration theory gear highlights five core elements for successful partnerships: Personnel, Knowledge, Trust, Process, and Resource. Effective collaboration is achieved by aligning these factors within a “Combined Framework,” illustrated as interconnected gears. The gears symbolize the complex, interdependent relationships and processes that enable a coordinated response

As mentioned above, integration and collaboration are independent but essential for resilient partnerships. Consequently, they need to be part of any constructive strategy that connects organizations. Having identified the need for inter-, intra-, and multidisciplinary integration and collaboration, and presenting gaps and common denominators between integration and collaboration theories, it appears logical to use a combined framework to enhance future DPHE management. This is based on the conducted critical integrative review. Figure 1 illustrates a conceptual framework for inter- and intra-organizational collaboration during emergencies, showing how different groups can integrate their efforts for a more effective and coordinated response. The framework is context-agnostic and applies to civil, military, and community actors across national settings. In countries where armed forces are not engaged domestically, the “military” gear should be interpreted as any organization providing scalable logistics, command, and surge capacity such as civil protection, gendarmerie, or national guard equivalents. Arrows connect the central gear to three main organizational types involved in a response, each represented with a distinct color and icon:

1. Healthcare organizations (blue gear): provide clinical expertise and medical care.
2. Military organizations (green gear): offer logistical support and command structures.
3. Community organizations (orange gear): contribute local knowledge and volunteer support.

These groups form a combined framework enabled by command and control, a shared communication model, and a common operational picture to harmonize their efforts.

#### The CSCATTT model

The CSCATTT model is presented as a linear process. This sequence represents the practical, operational steps taken during a disaster response. Each component represents a critical function: Command and Control, Safety, Communication, Assessment, Triage, Treatment, and Transport.

#### Expected outcomes

The final part of the diagram illustrates the expected outcomes of using this model. Successful implementation of the CSCATTT model and collaborative partnerships leads to:

1. Improved resource utilization – making the most of available resources.
2. Enhanced inter-agency coordination – better communication and teamwork between organizations.
3. Overall response effectiveness – achieving a more efficient and successful disaster response.

The effectiveness of collaboration depends on the event’s context, available support and resources, team and individual activities, roles, and tasks. The process is also influenced by overarching factors such as trust, interpersonal conflict, experience, goals, incentives, constraints, management, time, and performance. Using collaborative factors such as CSCATTT provides mutual points that facilitate coordination, cooperation, and ultimately collaboration. While coordination and cooperation are ideally established before an incident, strategies should exist to enable implementation immediately if necessary. The outcomes of collaboration can be measured through different methods. Using CSCATTT, quantitative, qualitative, and observational approaches have already shown results. The framework can be applied across different types of partnerships, particularly in civil–military interactions and EMT–community collaboration.

## Discussion

Disasters and public health emergencies (DPHE) challenge healthcare systems, often requiring additional resources and new measures in a complex, multidisciplinary management approach that addresses both natural and man-made hazards and includes risk assessments [2, 11, 12]. WHO recommends strengthening health systems and empowering communities through education and resource access to foster resilience. Building local capacity is crucial for long-term community resilience. Collaboration across sectors, including civil–military and public–private partnerships is also emphasized, as response effectiveness relies on the 4S of surge capacity: staff, supplies, space, and systems [71].

However, initial surges may require further expansion through community resources (FSC) and military assets, creating a collaborative pattern where integration between activities becomes essential. Collaboration requires understanding each entity’s limitations and potentials, coordinating efforts, and the testing of compatibility (cooperation). The goal is overlapping and seamless integration for effective partnerships, not full fusion or the dissolution of organizational boundaries and decision-making levels [5, 75]. Risk assessment, coordination, and cooperation should be initiated through interactions before an incident, enabling smoother collaboration. When different entities coordinate their resources and test their functionality, effective collaboration can be realized even during dynamic DPHE [34]. The synergy of integration and collaboration provides a holistic approach to disaster response, highlighting the importance of partnerships and education while also revealing potential gaps if collaboration is weak.

This study examined the possibility of combining integration and collaboration theories to develop a framework that supports stronger partnerships. The synthesis suggests that merging these theories can enhance teamwork and learning. Integration theory contributes by bringing together diverse resources and perspectives, while collaboration theory emphasizes collective efforts toward shared goals. Combined, they create inclusive environments that allow team members to benefit from a wide range of viewpoints and expertise [15, 25].

In practice, this combination strengthens problem-solving by providing teams with diverse information, defined roles, and accountability structures. Integration’s focus on resources and processes complements collaboration’s emphasis on flexibility and communication, fostering cohesive dynamics and responsiveness to changing conditions. Collaboration theory further stresses openness and knowledge-sharing, which combined with integration’s structural approach, can stimulate innovation and creativity [15, 25].

A further dimension is motivation and professional growth. By recognizing each member’s unique contribution, engagement and inclusiveness are promoted. Integration highlights the efficient use of resources, while collaboration emphasizes shared responsibilities. Together, they encourage effective resource utilization and continuous professional development as individuals refine competencies and adapt to new challenges in collaborative learning environments [15, 25].

These combined theories are not limited to healthcare. They can be applied across domains, including education and business, to strengthen teamwork, learning, and innovation. Overall, the synthesis suggests that integration and collaboration theories together foster environments where collaboration is more effective, learning more comprehensive, and problem-solving more innovative.

Beyond theoretical considerations, several recent studies illustrate the practical application of collaborative factors. Flexible resource management, community training, and collaborative networks have been instrumental in addressing disaster response challenges. For example, by integrating community activities with different partners and applying CSCATTT as the collaborative instrument volunteers were able to provide multiple services for suspected COVID-19 patients during a public health emergency in Bangkok, Thailand. The incident command system (ICS), embedded within the CSCATTT framework, standardized all CSCATTT components enabling a seamless integration of various teams with local authorities and other actors, reducing redundancy and supporting local recovery efforts [2, 11, 32–34, 76].

Technology further enhances collaboration. Mobile applications and geospatial mapping tools improve situational awareness and optimize resource allocation [68, 69]. Knowledge and information sharing are equally essential, allowing responders to adapt to the evolving nature of emergencies. Importantly, these technologies and the CSCATTT framework should be continuously tested in controlled environments where errors can be corrected without harm to victims.

Finally, training and education are consistently emphasized. Simulation exercises and public education have been shown to strengthen collaboration, facilitate FSC, and improve outcomes in DPHE response. These methods enhance readiness and allow responders to test and refine collaborative tools in realistic conditions [2, 11, 32, 33, 67, 77].

The core implications and practical use of this model can be demonstrated by lessons from two major events. First, Hurricane Katrina (2005): the widely criticized response highlighted the consequences of inadequate adaptive partnerships. Ineffective communication and poor coordination between federal, state, and local agencies caused significant delays in providing aid. Despite vast resources, the military was not effectively integrated into the public health response during the early phases. Second, the COVID-19 pandemic: this crisis demonstrated the urgent need for adaptive partnerships. Healthcare systems were overwhelmed, and partnerships with non-traditional actors became essential. Military organizations supported the establishment of field hospitals, vaccine distribution, and patient transportation. Community organizations contributed by disseminating information, assisting vulnerable populations, and managing food and supplies. In parallel, collaboration between public health agencies, private companies, and academic institutions accelerated the development and distribution of vaccines and treatments, a prime example of an adaptive partnership [55, 56].

Using this concept, effective emergency and disaster management extends beyond the actions of a single organization.

1. Breaking Down Silos: Traditional disaster response often isolates agencies in separate silos. The proposed framework emphasizes breaking these silos by fostering collaboration between healthcare systems, military organizations, and community groups. This enables a more holistic and less fragmented response.
2. Flexible and Scalable Response: A central feature of the framework is flexible surge capacity. Rather than relying on rigid plans, adaptive partnerships enable responses to scale up or down depending on crisis needs. Military logistics can transport medical supplies, while community volunteers can assist with non-clinical tasks, freeing healthcare professionals for essential duties.
3. Enhanced Communication and Coordination: The CSCATTT model provides standardized operational procedures that allow agencies to communicate and coordinate more effectively. This reduces confusion, accelerates decision-making, and improves response efficiency which is crucial for functions as triage and patient transport.
4. Community Resilience: Inclusion of community organizations ensures that responses are not only resource-efficient but also culturally sensitive and tailored to local needs. Communities are recognized not as passive victims but as active participants. Involving community-based organizations, volunteers, and local leaders that strengthens both immediate response and long-term recovery.

## Limitations

Unlike systematic reviews, which focus on narrowly defined questions and restrict the types of evidence considered, an integrative review draws on a broader range of sources to provide a holistic perspective and support theory development. Critical integrative reviews are particularly flexible, allowing synthesis of diverse types of literature, including experimental and non-experimental studies, as well as theoretical and empirical work. This breadth enables more comprehensive insights but also comes with limitations. High-quality integrative reviews require a transparent and rigorous process, including: 1) Formulating a clear research question or problem statement. 2) Conducting a systematic search to capture all relevant literature. 3) Critically analyzing and synthesizing findings across sources. In this study, these conditions were followed to minimize bias and ensure reliability. However, as with all integrative reviews, there remains a risk of selective interpretation. The proposed framework requires future validation, for example through a Delphi study with experts and by recurrent testing in simulation exercises.

## Conclusions

Combining integration and collaboration theories creates a strong synergy that enhances teamwork and learning. Integration theory brings together diverse resources and perspectives, enriching the environment for joint action. When combined with collaboration theory, which emphasizes working toward shared goals, this approach fosters a more inclusive and dynamic setting.

In such a model, valuing and integrating each team member’s unique perspective leads to richer discussions and more innovative solutions. Clear roles and responsibilities improve accountability and communication. Teams become more adaptable, creative, and efficient in resource use, while continuous learning becomes embedded in the culture.

The combined theoretical framework thus generates effective and innovative team dynamics that can be applied across multiple fields. It strengthens problem-solving, supports adaptability to evolving challenges, and fosters a sense of belonging and purpose among participants.

## Data Availability

All data used and cited in this study are available open source on the internet.

## Abbreviations

CSCATTT: Command and Control, Safety, Communication, Assessment, Triage, Treatment, and Transport
DPHE: Disasters and public health emergencies
EMT: Emergency medical teams
ICS: Incident Command System
WHO: World Health Organization
